# Biotechnological Prospects of Indigenous Oil‐Degrading Bacteria for Engine Oil Remediation and Environmental Restoration in Mekelle, Ethiopia

**DOI:** 10.1155/tswj/5366040

**Published:** 2026-03-17

**Authors:** Yohannes Tsegay Teklay, Desta Berhe Sbhatu, Gebreselema Gebreyohannes

**Affiliations:** ^1^ Faculty of Biotechnology, Mekelle Institute of Technology, Mekelle University, Mekelle, Ethiopia, mu.edu.et; ^2^ Faculty of Education, Universiti Teknologi MARA, Puncak Alam Campus, Puncak Alam, Selangor, Malaysia, uitm.edu.my

**Keywords:** bacteria, biodegradation, biosurfactants, Bushnell–Haas, engine oil, Mekelle

## Abstract

This study addressed environmental pollution from engine oil discharge in Mekelle’s workshops by isolating and characterizing oil‐degrading bacteria for bioremediation applications. Soil and water samples collected from 10 garage centers were analyzed for physicochemical properties, yielding six isolates: *Pseudomonas aeruginosa*, *Bacillus cereus*, *Staphylococcus aureus*, *Acinetobacter baumannii*, *Bacillus pumilus*, and *Bacillus megaterium*. Oil degradation assays on Bushnell–Haas agar with engine oil at 37°C for 14 days revealed that *B. pumilus* (98.9*%* ± 91.6*%*) and *A. baumannii* (98.7*%* ± 80*%*) were most effective in soil, while *B. megaterium* (96.9*%* ± 92.8*%*), *B. cereus* (96.7*%* ± 88.2*%*), and *P. aeruginosa* (96.5*%* ± 84.6*%*) excelled in water, with *B. megaterium* also showing high degradation in media (98.9*%* ± 88.8*%*). Biosurfactant production was strongest in *P. aeruginosa*, *A. baumannii*, and *B. cereus*; heavy metal tolerance was broad in *S. aureus* and *A. baumannii*; and salt tolerance was notable in *P. aeruginosa*, *B. megaterium*, *B. cereus*, and *B. pumilus*. Antibiotic susceptibility testing indicated that *P. aeruginosa*, *A. baumannii*, and *B. megaterium* could be safely applied in bioremediation, while resistance in *B. cereus*, *S. aureus*, and *B. pumilus* requires careful management. In conclusion, these isolates demonstrate strong potential as ecofriendly agents for oil spill remediation in Mekelle and similar environments.

## 1. Introduction

Engine oil contamination has become a critical global environmental challenge, particularly in urban areas where improper disposal from garages, workshops, and vehicles leads to extensive soil and water pollution [1]. Hydrocarbons persist in the environment, disrupting microbial diversity, reducing soil fertility, and posing serious risks to human health and ecosystems [2]. Conventional remediation methods, such as chemical dispersants and physical cleanup, are often costly, inefficient, and environmentally damaging [3]. These limitations have intensified interest in biotechnological approaches that harness the natural capabilities of microorganisms to degrade hydrocarbons in a sustainable and ecofriendly manner [4, 5].

With growing automobile use and industrial expansion, used engine oil poses a rising environmental burden, as lubricating oils accumulate toxic PAHs and heavy metals through heat and combustion [6]. When irresponsibly discarded through leaks, industrial discharge, or dumping, these contaminants infiltrate ecosystems, altering soil structure, polluting water sources, and bioaccumulating in plants and aquatic organisms [7]. The persistence of PAHs and heavy metals, coupled with weak environmental regulations in many regions, underscores the urgent need for robust and sustainable remediation strategies [8].

Indigenous bacteria offer unique advantages for bioremediation, especially in regions heavily impacted by oil pollution [9]. Prolonged exposure to local contaminants equips these microorganisms with specialized enzymes and metabolic pathways that enable them to degrade complex hydrocarbons more effectively than nonnative strains [10]. Their adaptation to local physicochemical conditions enhances survival and remediation efficiency. Many indigenous bacteria also produce biosurfactants that increase pollutant bioavailability, tolerate salinity and heavy metals and function synergistically in microbial consortia [11]. These traits make them promising candidates for restoring contaminated soils and waters, while also opening opportunities for industrial applications such as wastewater treatment, soil restoration, and biobased product development [12].

Bioremediation provides a low‐cost, ecofriendly way to combat oil pollution, using bacteria like *Pseudomonas* and *Bacillus* to enzymatically degrade hydrocarbons. Biosurfactants boost microbial access, while biostimulation and bioaugmentation enhance efficiency under optimal conditions of temperature, pH, and salinity [13]. Investigating indigenous oil‐degrading bacteria in Mekelle is vital for sustainable waste management, pollution mitigation, and ecosystem health, as it enables the use of native bacterial strains to remediate improper disposal of waste engine oil. Therefore, the objective of this study was to isolate, characterize, and evaluate indigenous oil‐degrading bacteria from engine oil–contaminated garage soils and waters in Mekelle for their potential application in bioremediation.

## 2. Materials and Methods

### 2.1. Sample Collection and Bacterial Isolation

Ten 30‐g soil samples were collected from oil‐contaminated workshops in Mekelle (10–12‐cm depth) using sterile trowels, with equal control samples taken from uncontaminated sites. Furthermore, 60‐mL water samples were collected from nearby oil‐contaminated rivers. All samples were labeled SS1, SS2, SS3, 17S1, 17S2, Ayn, Sem, Ku1, Ku2, and Ku3, based on their sources. Bacteria were subsequently isolated using serial dilution techniques [14]. In addition, 100 mL of fresh engine oil was obtained from oil distribution centers. The soil samples were sieved using a 2‐mm mesh to remove rocks and plant material and then homogenized. Finally, all samples were transported to the laboratory in an ice‐packed box and stored at −4°C for further analysis [15].

### 2.2. Physicochemical Properties of Soil Samples

Soil temperature was measured at 5–10‐cm depth using a thermometer, and the average was recorded [16]. Soil pH was measured using a calibrated pH meter in a slurry of dried, sieved soil and distilled water [17]. Soil moisture was determined gravimetrically by oven‐drying 5 g samples at 105°C for 24 h and calculating percentage mass loss [18]. Soil organic and inorganic content was measured by gravimetric analysis, with samples dried, sieved, and ignited at 550°C for 4 h; organic matter was calculated by difference, and inorganic content from mass loss [19].

### 2.3. Primary and Secondary Screening for Oil‐Degrading Bacteria

Soil dilutions were cultured in 100‐mL Bushnell–Haas broth with 5 mL engine oil and incubated in a shaker for 14 days [20]. Bushnell–Haas agar with engine oil and 0.1 mL bacterial culture was incubated at 37°C for 48 h, with controls lacking isolates. Colonies were transferred to nutrient agar, incubated 24 h, and cultured for morphological and biochemical characterization [21].

### 2.4. Characterization and Identification of Bacterial Isolates

Gram staining identified bacterial isolates by smearing, heat‐fixing, and sequentially staining with crystal violet, iodine, ethanol, and safranin; Gram‐positive appeared purple, and Gram‐negative pink under the microscope [22]. Endospore formation was assessed by staining heat‐fixed smears with malachite green, counterstaining with safranin, and observing green endospores within pink vegetative cells under the microscope [23].

Bacterial isolates were characterized using various biochemical tests, including the catalase test [24], motility test [25], indole test [26], Simmons citrate test [24], urease test [27], triple sugar iron (TSI) test [28], mannitol test [29], MacConkey test [30], and eosin methylene blue (EMB) test [31].

### 2.5. Determination of Engine Oil–Degrading Potential of Bacterial Isolates

Sterilized soil (10 g) and water (20 mL) were mixed with 5 mL engine oil and inoculated with 2 mL of each bacterial isolate after autoclaving at 121°C for 15 min. The mixtures were homogenized using a vortex. For the positive control, 10 g of sterilized soil and 10 mL of sterilized water were combined with 5 mL of fresh engine oil, and 2 mL of *P. aeruginosa* was inoculated. For the negative control, 10 g of sterilized soil and 10 mL of sterilized water were mixed with 5 mL of fresh engine oil without adding bacterial isolates. All tests and controls were incubated at 37°C for 14 days [32]. Six test tubes, each containing 50 mL of BH broth media, were prepared and mixed with 5 mL of fresh engine oil and 2 mL of each bacterial isolate. A positive control was prepared with fresh engine oil and a *P. aeruginosa* culture, while a negative control contained fresh engine oil without bacterial isolates. All test and control samples were incubated at 37°C for 14 days [33].

Soil, water, and media samples containing residual oil were collected at various intervals. Then, 10 mL of n‐hexane was added to each sample, mixed, and allowed to settle. The liquid phase was separated and collected in a beaker. The solvent was evaporated in an oven at 55°C. The residual oil was cooled, dissolved in methanol, and its optical density was measured using a spectrophotometer at 620 nm. Control samples were subjected to the same extraction process. The results were expressed as percentages of the absorbance of oil content in the samples over a period of 0–14 days [18, 24, 34, 35]. The percentage degradation of oil was calculated as follows:

Dergadation%=initial absorbance of oil−final absorbance of oilinitial absorbance×100%

### 2.6. Screening of Bacterial Isolates for Biosurfactant Production

The biosurfactant production ability of bacterial isolates was evaluated using several tests:

a. Drop collapse method: Engine oil was added to 96‐well microtiter plates and incubated for 1 h. Culture supernatant from six bacterial isolates was added to the oil layer, and the oil drop shape was examined after 1 min. Oil without bacterial culture served as a negative control. Flattened oil drops indicated positive biosurfactant production, while round drops indicated negative results [36].
b. Oil‐spreading method: Then, 20 *μ*L of oil was added to a plate containing 20 mL of distilled water, and 2 *μ*L of bacterial culture supernatant was applied to the oil‐covered water surface. The cleared zone was measured after 30 s. An area surrounded by emulsified brilliance indicated positive biosurfactant production [37].
c. Emulsification index method: Then, 5 mL of bacterial culture grown in nutrient broth was centrifuged, and the supernatant was collected. This supernatant was mixed with 3 mL of fresh engine oil, vortexed, and stored at room temperature for 24 h. The emulsion index was calculated as follows: *%*emulsification index = (height of the emulsified layer/total height of the liquid layer) × 100 [38].
d. Hemolytic activity: Blood agar, supplemented with 5% sheep blood, was incubated at 37°C for 24 h. The isolates were streaked onto six plates and examined for hemolysis. Beta‐hemolytic zones around colonies indicated biosurfactant production [39].

### 2.7. Screening of Bacterial Isolates for Heavy Metal Tolerance

A 100‐mM stock solution of metal salts, including zinc sulfate, copper sulfate, lead nitrate, iron chloride, and cobalt nitrate, was prepared by dissolving 0.3 g of each salt in 100 mL of distilled water. Dilutions of 1 mM, 5 mM, and 10 mM of each heavy metal were prepared from the stock solution. These dilutions were used for all metals, Zn, Cu, Pb, Fe, and Co. Test tubes containing 10 mL BH broth were supplemented with each metal concentration, and 100 *μ*L of overnight bacterial culture was inoculated into each test tube. The dilutions were then used to analyze the metal concentrations in the bacterial culture [40]. For the positive control, a test tube containing 10 mL of BH broth was supplemented with 100 *μ*L of *P. aeruginosa*, a bacterium known for its tolerance to heavy metals. A 10 mM solution of FeCl₂ was prepared to confirm the bacterium’s tolerance to heavy metals. For the negative control, a test tube containing 10 mL of BH broth was supplemented with a 10 mM solution of FeCl₂, but no bacterial culture was added. Test tubes were incubated at 37°C for 7 days, and growth of the inoculum was measured using spectrophotometer absorbance at 660 nm.

### 2.8. Screening of Bacterial Isolates for Salt Tolerance

Nutrient broths with varying concentrations of sodium chloride (NaCl) were prepared: 2 g of NaCl dissolved in 100 mL of nutrient broth for a 2% solution, 4 g for a 4% solution, 6 g for a 6% solution, and 8 g for an 8% solution. Each solution was autoclaved to ensure sterility. Subsequently, 100 *μ*L of overnight bacterial culture was inoculated into each broth using a sterile pipette. For the positive control, 100 *μ*L of *P. aeruginosa* culture, a clinically isolated bacterium known for its salt tolerance, was inoculated into each nutrient broth with different NaCl concentrations (2%, 4%, 6%, and 8%). For the negative control, 100 mL of nutrient broths with varying NaCl concentrations (2%, 4%, 6%, and 8%) were prepared and left uninoculated to ensure sterility and observe any changes without bacterial growth. All samples were incubated at 25C ± 2C for 5 days, and bacterial growth was monitored in comparison to the controls [41].

### 2.9. Screening of Bacterial Isolates for Compatibility Testing

Nutrient agar was supplemented with 5 mL of fresh engine oil. A bacterial isolate was streaked in a straight line at one end of the agar plate and incubated at 37°C for 24 h. This initial step exposed the first isolate to engine oil and set the stage for evaluating interactions between isolates. After the initial incubation, the remaining five isolates were streaked perpendicularly to the first isolate on the same plate. Each streak was incubated for an additional 24 h at 37°C. This procedure was repeated for each of the remaining five isolates, testing the newly cultured isolates against those previously cultured [42].

### 2.10. Antibiotic Susceptibility Test

The study used antibiotics like penicillin, imipenem, tetracycline, azithromycin, clindamycin, and cefoxitin (*μ*g/disc) to assess the resistance or susceptibility of different bacterial isolates. Mueller–Hinton agar plates were prepared for testing, with imipenem and tetracycline used for Gram‐negative bacteria and penicillin, azithromycin, clindamycin, and cefoxitin for Gram‐positive bacteria. Blank paper discs (6 mm) were used as controls. The plates were incubated for 24 h at 37°C, and the zones of inhibition were measured in millimeters [43]. The isolates were then categorized as susceptible, intermediate, or resistant based on standard guidelines such as the Clinical and Laboratory Standards Institute (CLSI) and the European Committee on Antimicrobial Susceptibility Testing (EUCAST) [44].

### 2.11. Data Analysis

The analysis was done using the new version of the SAS 9.4 software package, and results were presented as mean ± standard deviation. One‐way analyses of variance (ANOVAs) and post hoc analyses were also used to determine the significant differences between the means at *p* ≤ 0.05. Furthermore, the data was visualized using tables to facilitate interpretation and identify trends.

## 3. Results and Discussion

### 3.1. Physicochemical Properties of Soil Samples

Table 1 shows soil physicochemical properties across 10 sites, with temperatures (25°C–34°C) supporting microbial metabolism and oil degradation within the optimal 20°C–37°C range [45]. Soil pH ranged from 4.7 ± 0.2 to 6.6 ± 0.2, reflecting acidic to slightly acidic conditions conducive to oil‐degrading bacteria as studied by Yu et al. [46]. Moisture content varied between 1.0*%* ± 0.0*%* and 20*%* ± 0.2*%*, with values above 10% promoting microbial activity [47]. Organic matter was high, ranging from 75.9*%* ± 0.0*%* to 93.2*%* ± 0.5*%*, while inorganic content spanned 6.8*%* ± 0.2*%* to 24.1*%* ± 0.4*%*. Elevated organic matter supports microbial growth, whereas inorganic fractions influence oil degradation processes [48].

**Table 1:** Physicochemical properties of soil samples from Mekelle garage sites.

Soil samples	*T*(°C)	pH	MC (%)	OC (%)	IC (%)
SS1	26.0 ± 0.82	5.81 ± 0.1	12.40 ± 0.2	88.0 ± 1.0	12.0 ± 0.4
SS2	30.3 ± 2.01	5.20 ± 0.1	12.70 ± 0.1	83.2 ± 0.5	16.8 ± 0.2
SS3	27.0 ± 1.63	6.30 ± 0.0	10.68 ± 0.28	81.5 ± 0.3	18.5 ± 0.0
17S1	29.0 ± 0.82	6.19 ± 0.1	20.00 ± 0.2	76.5 ± 0.5	23.0 ± 0.2
17S2	31.0 ± 3.27	6.20 ± 0.2	7.04 ± 0.24	75.9 ± 0.0	24.1 ± 0.4
Ayn	26.8 ± 1.22	6.30 ± 0.1	8.88 ± 0.28	93.2 ± 0.5	6.80 ± 0.2
Sem	25.0 ± 3.27	5.85 ± 0.05	9.85 ± 0.15	85.4 ± 0.3	14.6 ± 0.0
Ku1	33.0 ± 4.08	4.70 ± 0.2	4.19 ± 0.19	90.9 ± 0.5	9.10 ± 0.0
Ku2	32.4 ± 2.45	6.50 ± 0.1	1.00 ± 0.0	86.5 ± 0.0	13.5 ± 0.2
Ku3	34.0 ± 0.00	6.60 ± 0.2	3.40 ± 0.2	85.3 ± 0.5	14.7 ± 0.4

*Note:* Values are expressed as mean ± SD.

Abbreviations: IC, inorganic content; MC, moisture content; OC, organic content; T, temperature.

### 3.2. Morphological and Biochemical Characterization of Bacterial Isolates

**Figure 1:**
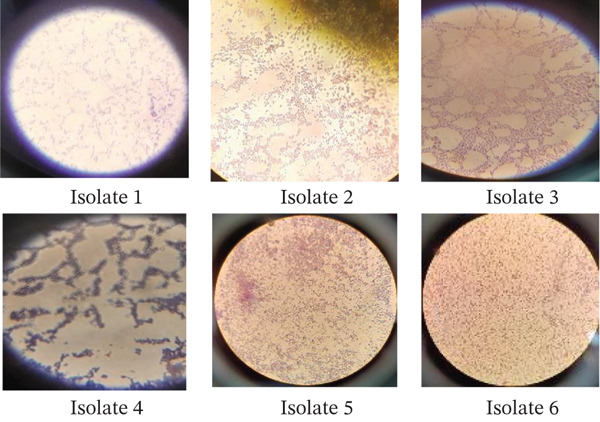
Microscopic appearance of G‐stained bacterial isolates. Key: Isolate 1 = SS1; Isolate 2 = Ayn; Isolate 3 = SS2; Isolate 4 = Sem; Isolate 5 = 17s1; and Isolate 6 = SS3.

Six isolates, namely, SS‐1, SS‐2, SS‐3, 17‐s1, 17‐s2, and Ayn were isolated with the colony count number (Log_10_CFU) of 96 ± 1, 112 ± 0, 139 ± 0, 133 ± 2, 68 ± 0, and 123 ± 2, respectively (Table 2). Based on the morphological, biochemical, and G‐staining analysis and by taking *Bergey’s Manual of Determinative Bacteriology* as a reference, the bacterial isolates were identified into their respective species. The SS1, SS‐2, SS‐3, 17‐s1, Ayn, and Sem were identified as *Bacillus cereus*, *Staphylococcus aureus, Acinetobacter baumannii*, *Pseudomonas aeruginosa*, *Bacillus megaterium*, and *Bacillus pumilus*, respectively (Figure 1).

**Table 2:** G stain and morphological characteristics of six bacterial isolates.

Isolates	G stain	Colony color	Cell shape
17s1	−ve	Pink	Cocci
SS1	+ve	Purple	Rod
Ayn	+ve	Purple	Rod
SS2	+ve	Purple	Cluster cocci
SS3	−ve	Pink	Cocci
Sem	+ve	Purple	Cocci chain

Abbreviations: 17s1, Isolate 1; Ayn, Isolate 3; G−ve, G negative; G + ve, G positive; Sem, Isolate 6; SS1, Isolate 2; SS2, Isolate 4; SS3, Isolate 5.

Biochemical profiling revealed distinct traits among the isolates (Table 3). 17s1 was catalase‐positive, H₂S‐ and citrate‐producing, mannitol‐ and MacConkey‐positive, with a K/K TSI and dark EMB precipitate. SS1 was catalase‐ and urease‐positive, spore‐forming, motile, citrate‐positive, with purple EMB colonies and an A/A TSI. Ayn was catalase‐positive, spore‐forming, mannitol‐positive, citrate‐negative, with clear EMB colonies and k/k TSI. SS2 was catalase‐ and urease‐positive, mannitol‐positive, citrate‐negative, with clear EMB colonies and A/K TSI. SS3 was catalase‐positive, motile, citrate‐ and gas‐producing, MacConkey‐positive, with dark EMB precipitate and A/A TSI. Sem was catalase‐ and urease‐positive, motile, citrate‐ and gas‐producing, mannitol‐positive, MacConkey‐negative, with purple EMB colonies and A/A TSI.

**Table 3:** Biochemical characteristics of bacterial isolates.

Biochemical test			Isolates			
	17s1 *(P. aeruginosa)*	SS1 (*B. cereus)*	Ayn (*B. megaterium)*	SS2 (*S. aureus)*	SS3 (*A. baumannii)*	Sem (*B. pumilus)*
Catalase test	+	+	+	+	+	+
Urease test	−	+	−	+	−	+
Indole test	−	−	−	−	−	−
Spore formation	−	+	+	−	−	−
Motility	−	+	−	−	+	+
H_2_S production	+	−	−	−	−	−
Citrate	+	+	−	−	+	+
Gas production	−	−	−	−	+	+
Eosin Methylene Blue test	DP	PC	C	C	DP	P
Mannitol	+	−	+	+	−	+
MacConkey	+	−	−	−	+	+
Triple sugar iron test	K/K	A/A	k/k	A/K	A/A	A/A

Abbreviations: (**+**), positive; (−), negative; A/A, glucose, lactose, and sucrose fermentation; A/K, glucose fermentation; C, colorless; DP, dark purple; k/k, no fermentation; P, purple; PC, purple and colorless.

### 3.3. Determination of Engine Oil–Degrading Potential of Isolates

**Figure 2:**
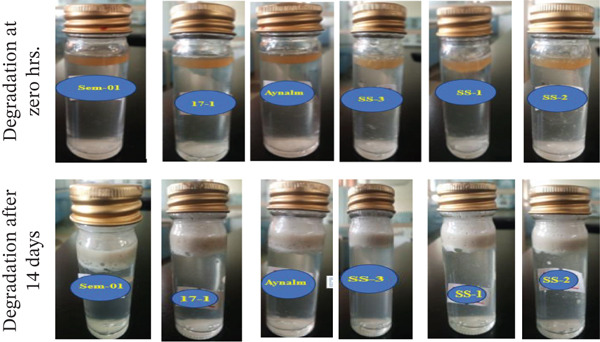
Degradation ability of bacterial isolates on fresh engine oil in water.

**Figure 3:**
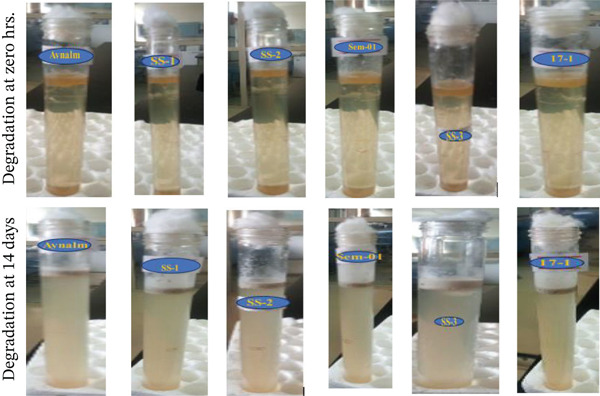
Degradation ability of bacterial isolates on fresh engine oil in BH media.

**Figure 4:**
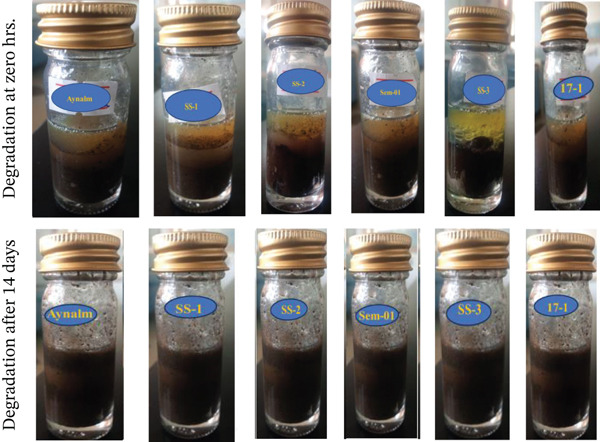
Degradation ability of bacterial isolates on fresh engine oil in soil.

The study found that *B. cereus*, *S. aureus*, *A. baumannii*, *B. pumilus*, *B. megaterium*, and *P. aeruginosa* have the potential to degrade engine oil in soil, water, and media (Figures 2, 3, and 4), which aligns with previous research done by Al‐Dhabaan [24]. Oil degradation was uniform at 0 h, but by 24 h absorbance differences revealed varying bacterial potential. Early rates were low, likely due to bacterial adaptation to oil [49]. By 14 days, degradation differences were pronounced, with near‐complete oil breakdown, indicating bacterial adaptation to hydrocarbons as a carbon source [50].

*B. cereus* achieved high biodegradation in soil (97.3*%* ± 87.0*%*), media (94.2*%* ± 85.7*%*), and water (96.7*%* ± 88.2*%*), confirming its potent hydrocarbon‐degrading enzymes [24, 51] (Table 4). Its adaptability across environments highlights its promise for cost‐effective bioremediation [52, 53]. *S. aureus* exhibited the highest degradation in water (95.3*%* ± 91.6*%*), followed by soil (93.2*%* ± 90.0*%*) and media (92.6*%* ± 66.7*%*), consistent with earlier findings [54, 55]. *A. baumannii* showed maximum degradation in soil (98.7*%* ± 80.0*%*) and strong activity in water (94.6*%* ± 92.3*%*), reflecting its alkane hydroxylase enzymes and resilience in extreme conditions [56, 57]. *B. pumilus* degraded oil most effectively in soil (98.9*%* ± 91.6*%*) but less in water (80.9*%* ± 78.2*%*), indicating mechanisms optimized for solid matrices [58, 59].

**Table 4:** Biodegradation of engine oil by bacterial isolates across different conditions over 14 days.

Condition	Isolates	0 h	24 h	7 days	14 days	Degradation (%)
Soil	*B. cereus*	0.97 ± 0.89^a^	0.75 ± 0.12^b^	0.4 ± 0.04^c^	0.02 ± 0.02^a^	97.9 ± 0.87.
	*S. aureus*	0.90 ± 0.30^a^	0.70 ± 0.16^a^	0.3 ± 0.08^d^	0.06 ± 0.03^b^	93.2 ± 0.90
	*A. baumannii*	0.96 ± 0.05^a^	0.69 ± 0.03^a^	0.34 ± 0.01^c^	0.01 ± 0.01^c^	98.7 ± 0.80
	*B. pumilus*	0.95 ± 0.12^a^	0.63 ± 0.10^c^	0.23 ± 0.06^a^	0.01 ± 0.01^c^	98.9 ± 0.91
	*B. megaterium*	0.85 ± 0.00^a^	0.58 ± 0.06^b^	0.34 ± 0.01^c^	0.04 ± 0.02^b^	95.3 ± 0.75
	*P. aeruginosa*	0.99 ± 0.15^a^	0.71 ± 0.08^b^	0.37 ± 0.05^c^	0.12 ± 0.01^d^	87.9 ± 0.93
Water	*B. cereus*	0.92 ± 0.17^a^	0.85 ± 0.12^b^	0.52 ± 0.09^c^	0.03 ± 0.02^d^	96.7 ± 0.88
	*S. aureus*	0.85 ± 0.12^a^	0.75 ± 0.08^a^	0.43 ± 0.02^c^	0.04 ± 0.01^a^	95.3 ± 0.91
	*A. baumannii*	0.93 ± 0.26^a^	0.8 ± 0.16^ab^	0.35 ± 0.12^a^	0.05 ± 0.02^b^	94.6 ± 0.92
	*B. pumilus*	0.89 ± 0.23^a^	0.8 ± 0.16^ab^	0.6 ± 0.08^ca^	0.17 ± 0.05^c^	80.9 ± 0.78
	*B. megaterium*	0.98 ± 0.14^a^	0.77 ± 0.13^a^	0.43 ± 0.10^a^	0.03 ± 0.01^d^	96.9 ± 0.92
	*P. aeruginosa*	0.87 ± 0.13^a^	0.37 ± 0.09^ab^	0.19 ± 0.07^c^	0.03 ± 0.02^a^	96.5 ± 0.85
Media	*B. cereus*	0.87 ± 0.14^a^	0.75 ± 0.12^b^	0.6 ± 0.08^a^	0.05 ± 0.02^a^	94.2 ± 0.86
	*S. aureus*	0.95 ± 0.12^a^	0.82 ± 0.09^b^	0.5 ± 0.00^b^	0.07 ± 0.04^c^	92.6 ± 0.67
	*A. baumannii*	0.87 ± 0.14^a^	0.60 ± 0.08^b^	0.2 ± 0.05^d^	0.11 ± 0.01^d^	87.3 ± 0.93
	*B. pumilus*	0.94 ± 0.11^a^	0.80 ± 0.08^bc^	0.40 ± 0.04^e^	0.09 ± 0.03^b^	90.4 ± 0.73
	*B. megaterium*	0.92 ± 0.09^a^	0.45 ± 0.04^b^	0.23 ± 0.02^c^	0.01 ± 0.01^c^	98.9 ± 0.89
	*P. aeruginosa*	0.91 ± 0.08^a^	0.65 ± 0.04^c^	0.34 ± 0.03^f^	0.09 ± 0.02^d^	90.1 ± 0.75
	+ve control	0.98 ± 0.00^a^	0.05 ± 0.00^b^	0.02 ± 0.00^c^	0.00 ± 0.00^d^	100 ± 0.00

*Note:* Values of the same column followed by different letters are significantly different at *p* ≤ 0.05.

*B. megaterium* demonstrated consistent degradation in soil (95.3*%* ± 75.0*%*), water (96.9*%* ± 92.8*%*), and media (98.9*%* ± 88.8*%*), supported by extensive enzymatic pathways and nutrient supplementation [60]. *P. aeruginosa* showed lower efficiency in soil (87.9*%* ± 93.3*%*) but higher in water (96.5*%* ± 84.6*%*) and media (90.1*%* ± 75.0*%*), aligning with its adaptation to aqueous environments [61]. Its combination with *B. cereus* and *B. megaterium* could strengthen bioremediation strategies for Mekelle’s oil‐contaminated ecosystems.

### 3.4. Screening of Bacterial Isolates for Biosurfactant Production

Figure 5 shows that *P. aeruginosa*, *B. cereus*, and *S. aureus* formed flat drops in the drop‐collapsing test, indicating strong biosurfactant activity linked to rhamnolipids and lipopeptides [62, 63]. This activity enhances oil degradation by increasing pollutant availability and reducing surface tension. In contrast, *B. megaterium*, *B. pumilus*, and *A. baumannii* produced round drops, reflecting lower biosurfactant production and reduced degradation potential [62, 63].

Figure 5Comparative drop collapsing test of six bacterial isolates and a positive control. Key: (a) before and (b) after; 17s1 = *P. aeruginosa*; SS1 = *B. cereus*; SS2 = *S. aureus*; SS3 = *A. baumannii*; Sem = *B. pumilus*; and Ayn = *B. megaterium.*

**Figure:**
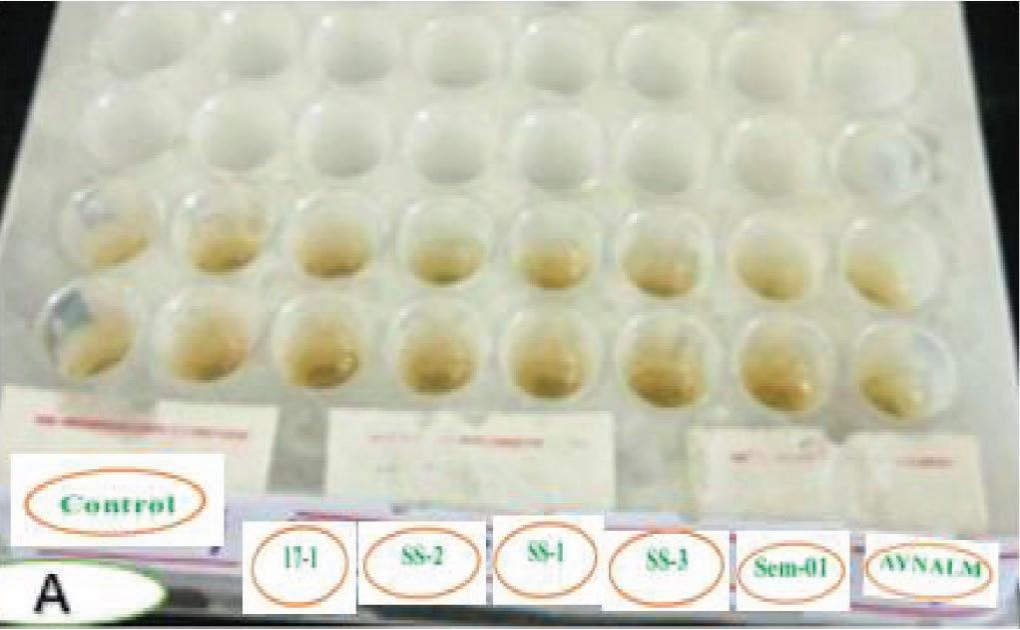
(a)

**Figure:**
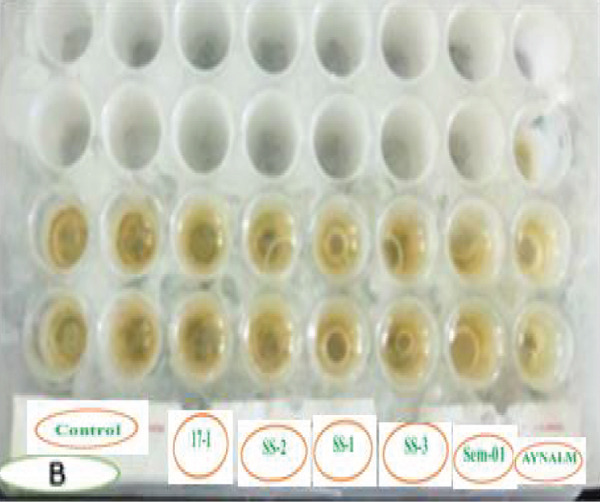
(b)

Figure 6 highlights differences in oil displacement among isolates. *P. aeruginosa* showed the highest activity (5 ± 1.15 cm), reflecting strong surfactant potential from rhamnolipid production [64, 65], making it a promising candidate for oil spill bioremediation. *B. cereus* displayed moderate displacement (3 ± 0.82 cm), likely linked to lipopeptide production, useful for targeted remediation where moderate surfactant activity suffices [66]. *B. megaterium* exhibited limited spreading (1.5 ± 0 cm), indicating lower emulsification capacity and reduced biosurfactant output. In contrast, *S. aureus*, *A. baumannii*, and *B. pumilus* showed no displacement, suggesting minimal biosurfactant activity under these conditions, despite reports of production in certain strains [67–69]. Generally, he observed displacement in *P. aeruginosa*, *B. cereus*, and *B. megaterium* confirms biosurfactant production, critical for engine oil bioremediation in Mekelle [69–71].

Figure 6Oil‐spreading ability of the bacterial isolates, where (a) before and (b) after.

**Figure:**
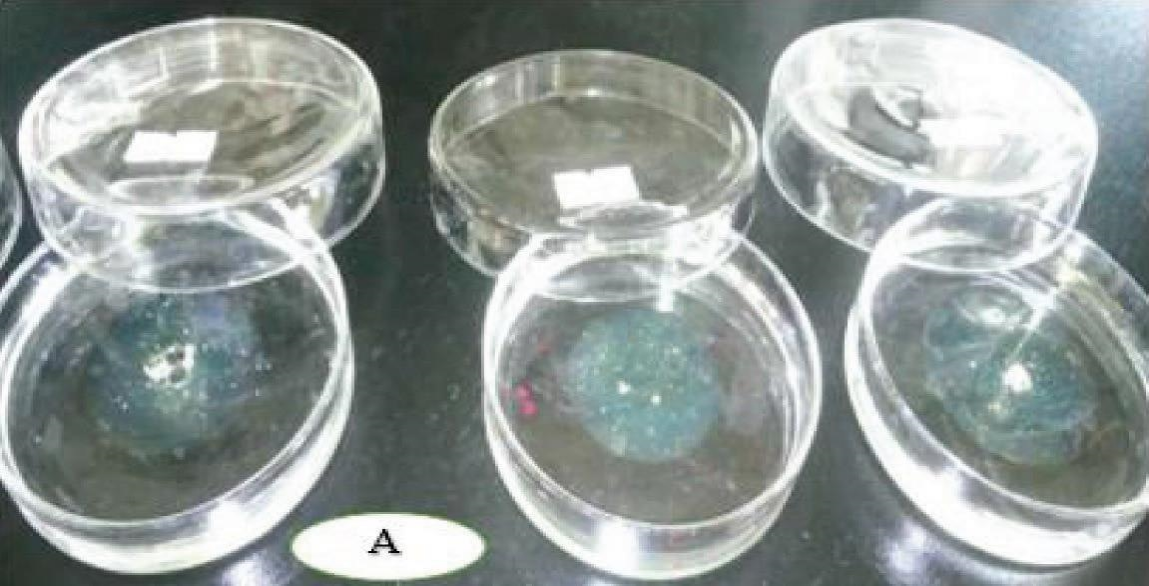
(a)

**Figure:**
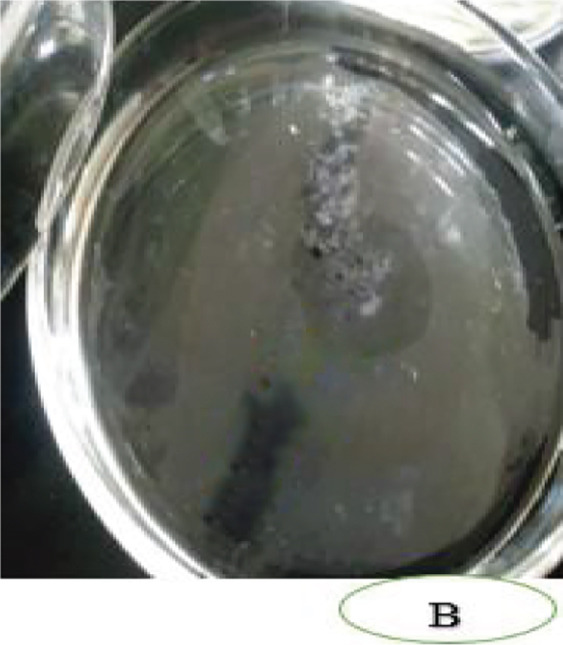
(b)

**Figure 7:**
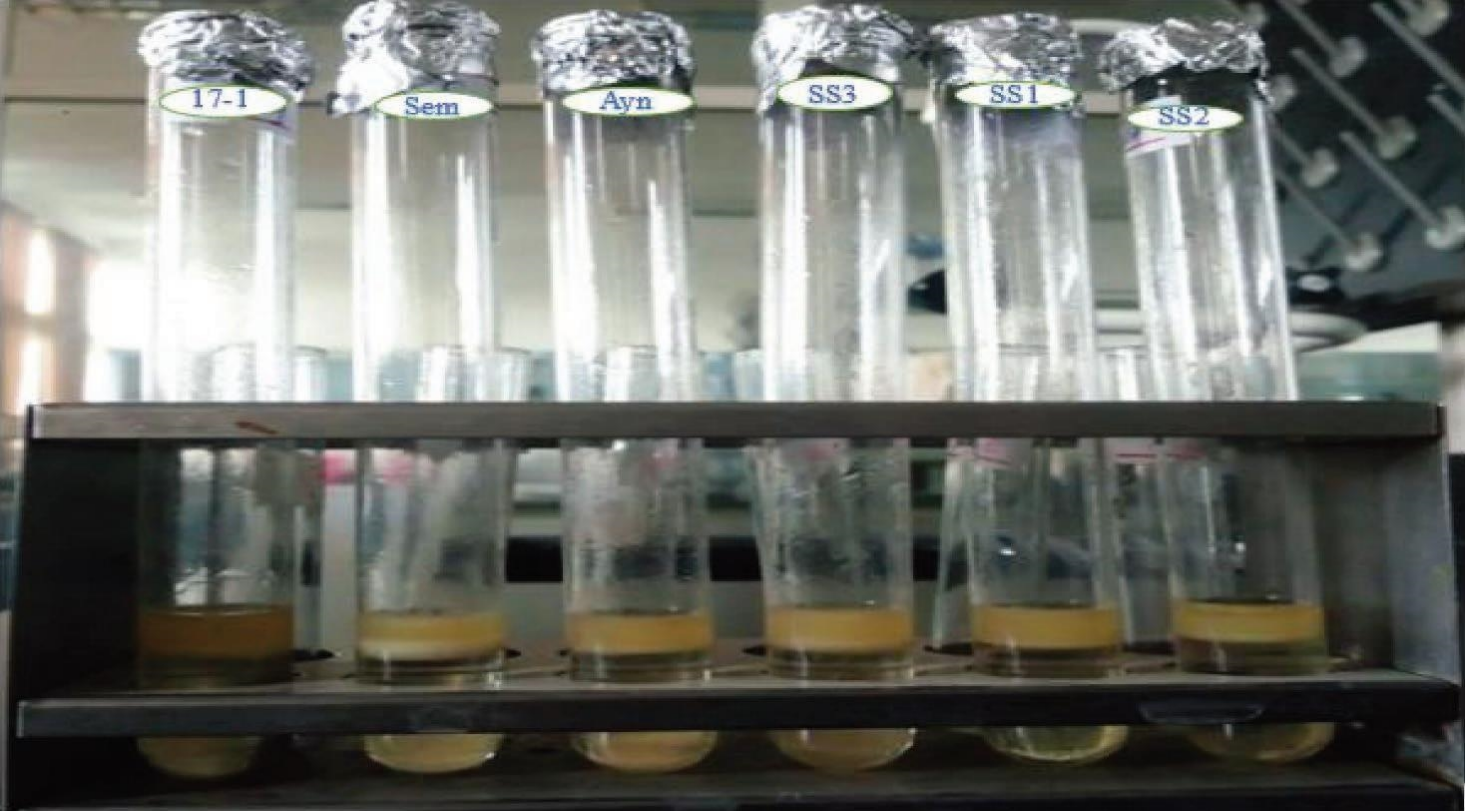
Emulsification stability test of isolates. Key: 17s1 = *P. aeruginosa*; Sem = *B. pumilus*; Ayn = *B. megaterium*; SS3 = *A. baumannii*; SS1 = *B. cereus*; and SS2 = *S. aureus.*

*A. baumannii* (23.9*%* ± 0.9*%*) and *S. aureus* (21.3*%* ± 2*%*) exhibited high emulsifying activity, indicating strong potential for stabilizing emulsions and enhancing bioremediation (Figure 7 and Table 5). *P. aeruginosa* demonstrated moderate stability (18*%* ± 3.0*%*), consistent with its role as a prolific rhamnolipid producer (Radzuan et al. [72]. *B. pumilus* also showed moderate activity (17.8*%* ± 1.8*%*) (Patowary et al. [59], while *B. cereus* displayed low stability (3.03*%* ± 2.69*%*), reflecting strain variability, though *Bacillus* species are known to produce lipopeptides such as surfactin (Abdelraof et al. [73].

**Table 5:** Biosurfactant and hemolytic activities of bacterial isolates.

Bacterial isolates	Shape of drop	Oil spreading	Emulsification stability (%)	Hemolytic activity
*P. aeruginosa*	Flat	5 ± 1.15	18 ± 3.0	*β*
*B. cereus*	Flat	3 ± 0.82	3.03 ± 2.69	*γ*
*B. megaterium*	Round	1.5 ± 0.0	21.21 ± 1.22	*α*
*S. aureus*	Flat	—	21.3 ± 2.0	*β*
*A. baumannii*	Round	—	23.9 ± 0.9	*γ*
*B. pumilus*	Round	—	17.8 ± 1.8	*γ*

*Note:* Values expressed as mean ± SD.

Abbreviations: *α*, alpha; *β*, beta; *γ*, gamma.

**Figure 8:**
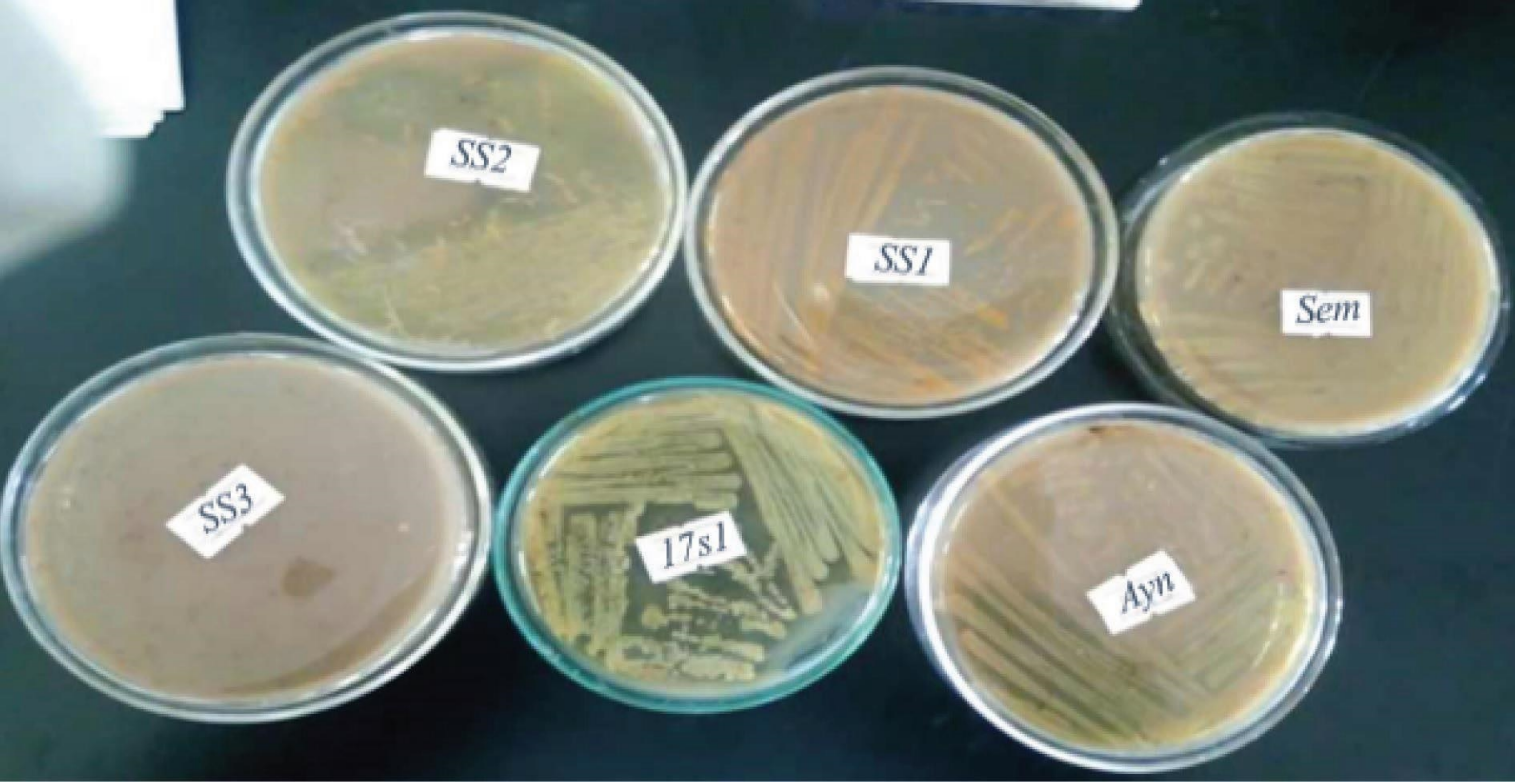
Hemolytic activity of six bacterial isolates. *Key:* 17s1 = *P. aeruginosa*; SS1 = *B. cereus*; SS2 = *S. aureus*; SS3 = *A. baumannii*; Sem = *B. pumilus*; and Ayn = *B. megaterium.*

*P. aeruginosa* and *S. aureus* exhibited complete (*β*) hemolysis, demonstrating virulence associated with potent biosurfactants such as rhamnolipids and staphylococcal beta‐hemolysin (Liu et al., [74] (Figure 8 and Table 5). While these compounds enhance bioremediation by reducing interfacial tension and stabilizing emulsions, their pathogenicity necessitates careful monitoring to balance ecological benefits with health risks. In contrast, *γ*‐hemolytic strains (*B. cereus*, *B. pumilus*, and *A. baumannii*) produced effective biosurfactants with lower pathogenicity, making them safer candidates for oil spill remediation. *B. megaterium* displayed *α*‐hemolysis, indicating moderate pathogenicity but also industrial relevance through enzyme and biosurfactant production. In general, all isolates produced biosurfactants, with *P. aeruginosa*, *A. baumannii*, and *B. cereus* showing the greatest promise. The observed variations highlight the need for multiple screening approaches to fully evaluate biosurfactant potential.

### 3.5. Screening of Bacterial Isolates for Heavy Metal Tolerance

Optical density at 660 nm showed variable heavy metal tolerance, with *P. aeruginosa* and *A. baumannii* most resilient, while *S. aureus*, *B. pumilus*, and *B. megaterium* displayed reduced growth at higher concentrations (Table 6). The study revealed that *B. cereus* exhibits selective tolerance to zinc, lead, and cobalt at concentrations of 1, 5, and 10 mM, consistent with earlier findings ([75]; [76]). This selective tolerance highlights its potential role in bioremediation of environments contaminated with these metals, particularly oil‐polluted soils where bioaccumulation may aid restoration [77]. However, its intolerance to copper and iron limits its application in more complex contamination scenarios, requiring complementary microorganisms to address broader heavy metal pollution. In contrast, *S. aureus* demonstrated broad tolerance to all tested metals, supported by mechanisms such as metallothionein production and efflux pumps [78], making it highly adaptable for remediation in mixed‐pollutant environments. Similarly, *B. pumilus* tolerated zinc, copper, lead, and iron but was sensitive to cobalt, while *B. megaterium* showed tolerance to zinc, copper, and iron but not cobalt or lead, indicating their selective utility in targeted remediation contexts.

**Table 6:** Heavy metal tolerance of bacterial isolates at different concentrations.

Conc (mM)	Bacterial isolates	Optical density of heavy metals at 660 nm				
		ZnSO_4_	CuSO_4_	Pb (NO3)_2_	FeCl_2_	Co (NO_3_)
1	*B. cereus*	1.2 ± 0.2^a^	0.3 ± 0.2^a^	1.1 ± 0.2^a^	0.2 ± 0.0^a^	1.3 ± 0.3^a^
	*S. aureus*	1.3 ± 0.3^b^	1.2 ± 0.1^a^	1.4 ± 0.2^c^	1.1 ± 0.0^a^	1.3 ± 0.3^e^
	*A. baumannii*	1.4 ± 0.2^a^	1.3 ± 0.1^b^	1.3 ± 0.0^b^	1.2 ± 0.2^c^	1.4 ± 0.2^a^
	*B. pumilus*	1.3 ± 0.3^a^	1.2 ± 0.1^a^	1.1 ± 0.2^c^	1.0 ± 0.2^b^	0.3 ± 0.1^a^
	*B. megaterium*	1.2 ± 0.1^a^	1.1 ± 0.2^b^	0.2 ± 0.0^d^	1.2 ± 0.2^d^	0.4 ± 0.1^b^
	*P. aeruginosa*	1.4 ± 0.2^b^	1.3 ± 0.1^a^	1.4 ± 0.0^e^	1.3 ± 0.0^e^	1.4 ± 0.2^c^
5	*B. cereus*	1.0 ± 0.1^ab^	0.1 ± 0.0^ab^	0.9 ± 0.2^a^	0.1 ± 0.0^ac^	1.0 ± 0.0^a^
	*S. aureus*	1.1 ± 0.1^b^	1.0 ± 0.1^c^	1.2 ± 0.1^b^	0.9 ± 0.0^ab^	1.1 ± 0.2^b^
	*A. baumannii*	1.2 ± 0.1^c^	1.1 ± 0.1^a^	1.1 ± 0.0^c^	0.7 ± 0.2^bc^	1.1 ± 0.1^c^
	*B. pumilus*	1.1 ± 0.2^bc^	1.1 ± 0.2^b^	1.1 ± 0.2^c^	1.1 ± 0.2^a^	1.1 ± 0.2^d^
	*B.megaterium*	1.0 ± 0.0^a^	1.0 ± 0.0^d^	1.0 ± 0.0^d^	1.0 ± 0.0^b^	1.0 ± 0.0^e^
	*P. aeruginosa*	0.9 ± 0.1^c^	0.9 ± 0.1^e^	0.9 ± 0.1*e*	0.9 ± 0.1^c^	0.9 ± 0.1^f^
10	*B. cereus*	0.8 ± 0.0^a^	0.8 ± 0.0^ab^	0.8 ± 0.0^a^	0.8 ± 0.0^ab^	0.8 ± 0.0^dc^
	*S. aureus*	0.0 ± 0.0^b^	0.0 ± 0.0^b^	0.0 ± 0.0^c^	0.0 ± 0.0^ac^	0.0 ± 0.0^a^
	*A. baumannii*	0.7 ± 0.2^c^	0.7 ± 0.2^b^	0.7 ± 0.2^c^	0.7 ± 0.2^ad^	0.7 ± 0.2^b^
	*B. pumilus*	0.0 ± 0.0^c^	0.0 ± 0.0^c^	0.0 ± 0.0^d^	0.0 ± 0.0^b^	0.0 ± 0.0^c^
	*B.megaterium*	0.8 ± 0.1^d^	0.8 ± 0.1^c^	0.8 ± 0.1^b^	0.8 ± 0.1^c^	0.8 ± 0.1^d^
	*P. aeruginosa*	0.8 ± 0.0^e^	0.8 ± 0.0^ab^	0.8 ± 0.0^d^	0.8 ± 0.0^d^	0.8 ± 0.0^e^
	+ve control	1.4 ± 0.2^a^	1.3 ± 0.1^b^	1.4 ± 0.2^c^	1.2 ± 0.2^d^	1.1 ± 0.2^e^

*Note:* Values of the same column followed by different letters are significantly different at *p* ≤ 0.05.

*P. aeruginosa* stood out as a highly effective agent, displaying broad tolerance, rapid adaptation, and superior metal removal efficiency through biosorption and bioaccumulation ([79–82]; R. [83]). Its resilience in oil‐contaminated and metal‐polluted environments underscores its promise for bioremediation in Mekelle. In general, the diverse tolerance profiles of the isolates support a targeted strategy: broad‐spectrum strains such as *S. aureus*, *A. baumannii*, and *P. aeruginosa* are suited for complex contamination, while selective strains like *B. cereus*, *B. pumilus*, and *B. megaterium* are best applied in specific contexts. Combining these organisms into microbial consortia could maximize remediation efficiency, offering cost‐effective and sustainable solutions for heavy metal and oil pollution.

### 3.6. Screening of Bacterial Isolates for Salt Tolerance

The six bacterial isolates displayed varying tolerance to NaCl (Table 7). *P. aeruginosa* grew at 2%, 4%, and 6% NaCl but failed at 8%, indicating a tolerance limit of 6%. Previous studies reported optimal crude oil degradation by *Pseudomonas* at mild salinity (1% NaCl) and highlighted its resilience under alkaline conditions [84, 85]. This suggests strong potential for bioremediation in saline environments of Mekelle, where other microbes may not survive. In contrast, *B. cereus* and *B. megaterium* thrived only at lower NaCl levels but failed above 4%, consistent with reports of growth delays and reduced rates under elevated salinity [86]. Their restriction to low‐salinity conditions limits application in highly saline sites, underscoring the need for careful salinity management to optimize oil degradation [87].

**Table 7:** Salt tolerance of bacterial isolates at varying NaCl concentrations.

Bacterial isolates		Salt concentration		
	2%	4%	6%	8%
*B. cereus*	+	+	+	−
*S. aureus*	+	−	−	−
*A. baumannii*	+	−	−	−
*B. pumilus*	+	+	+	−
*B. megaterium*	+	+	+	−
*P. aeruginosa*	+	+	+	−

*Note:* ‘+’ denotes the presence of growth, whereas ‘−’ denotes the absence of growth.

*A. baumannii* and *S. aureus* showed enhanced growth at 2%–6% NaCl, while *B. pumilus* tolerated up to 6%, reflecting higher salt resilience [88, 89]. This adaptability makes them suitable for bioremediation in oil‐contaminated areas with variable salinity. Their hydrocarbon‐degrading capacity aligns with previous findings that *Pseudomonas*, *Bacillus*, *Acinetobacter*, and *Staphylococcus* species can function effectively in saline environments [90–92]. Collectively, these salt‐tolerant strains offer promising tools for restoring Mekelle’s ecosystems, ensuring efficient oil degradation while maintaining microbial functionality.

### 3.7. Screening of Bacterial Isolates for Compatibility Testing

**Figure 9:**
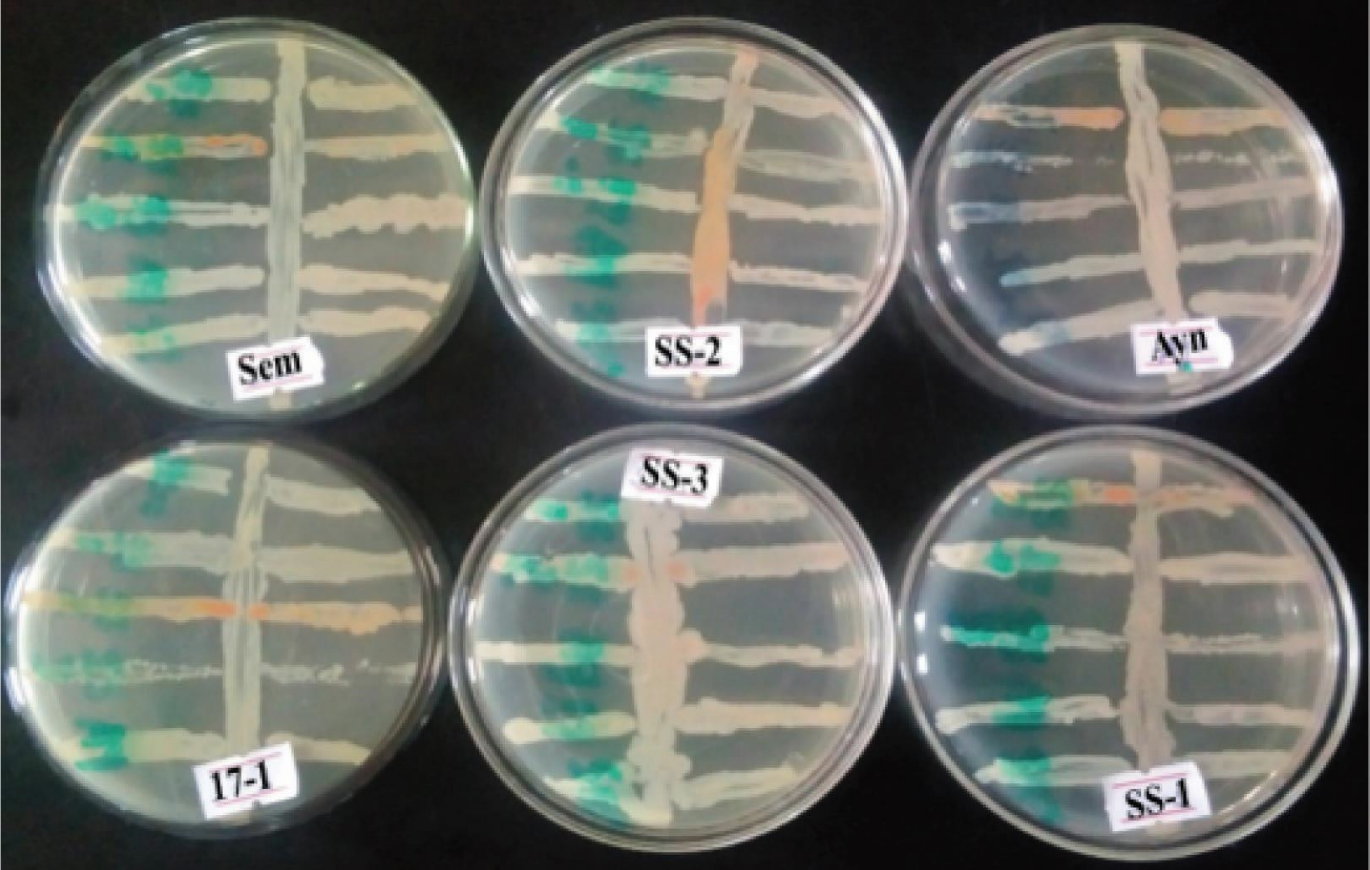
Compatibility assessment of six bacterial isolates. Key: Sem = *B. pumilus*; SS2 = *S. aureus*; Ayn = *B. megaterium*; 17s1 = *P. aeruginosa*; SS3 = *A. baumannii*; and SS1 = *B. cereus*.

Six bacterial isolates demonstrated synergistic bioremediation potential in co‐culture, degrading hydrocarbons more efficiently than individual strains through diverse metabolic pathways, thereby supporting the development of a robust consortium for environmental cleanup (Figure 9). Their ability to colonize oil‐contaminated soils offers a green, ecofriendly solution for remediation in Mekelle, where oil pollution has adversely affected agriculture and communities [93], and harnessing these isolates could promote sustainable restoration while mitigating the impacts of contamination.

### 3.8. Antibiotic Susceptibility Test

**Figure 10:**
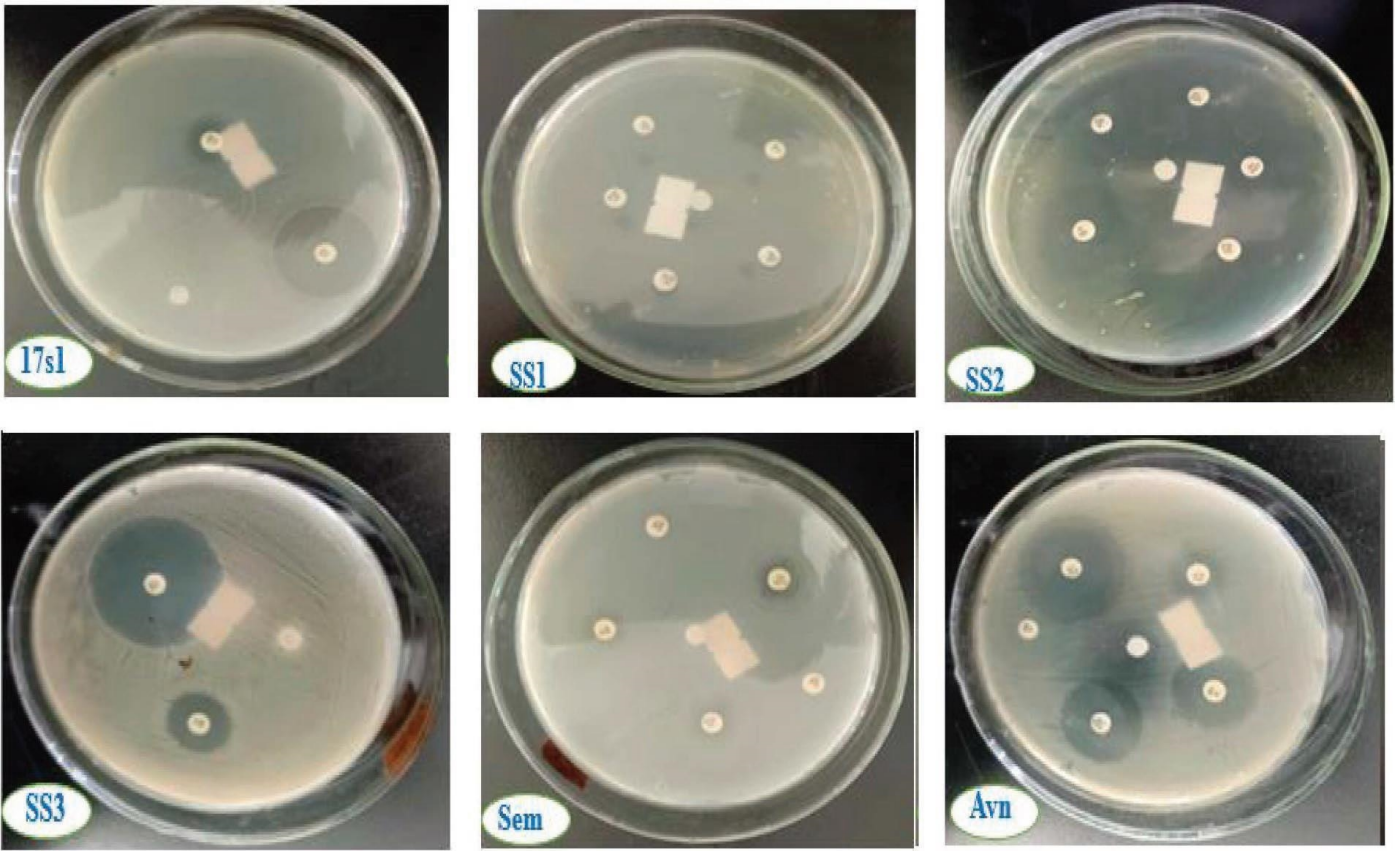
Antibiotic susceptibility test of bacterial isolates in terms of inhibition zone. Key: 17s1 = *P. aeruginosa*; SS1 = *B. cereus*; SS2 = *S. aureus*; SS3 = *A. baumannii*; Sem = *B. pumilus*; and Ayn = *B. megaterium.*

Antibiotic sensitivity testing revealed distinct resistance and susceptibility patterns among the isolates (Figure 10 and Table 8). *P. aeruginosa* was highly susceptible to imipenem (39 ± 13 mm) but resistant to tetracycline (17 ± 6.0 mm), consistent with earlier findings [94]. The significant difference in inhibition zones (*p* < 0.05) highlights imipenem as a suitable treatment option, while tetracycline remains ineffective. Despite its pathogenic potential, *P. aeruginosa* could be applied in bioremediation of oil‐contaminated sites in Mekelle, provided antibiotic risks are carefully managed. Similarly, *A. baumannii* showed resistance to tetracycline but selective sensitivity to imipenem (Pourhajibagher et al. [95], supporting its use in remediation when paired with appropriate antibiotic control.

**Table 8:** Antibiotic susceptibility of bacterial isolates.

Isolates		Inhibition zone (mm)				
	Fox	PG10	CD2	Ath	T30	IMI
*P. aeruginosa*	—	—	—	—	17 ± 6.0^e^	39 ± 13^a^
*B. cereus*	25 ± 4.0^a^	12 ± 0.0^a^	4.0 ± 0.0^a^	29 ± 10^a^	20 ± 8.0^a^	—
*B. megater*	30 ± 0.0^a^	34 ± 20^b^	25 ± 0.0^b^	26 ± 15^b^	22 ± 13^b^	—
*S. aureus*	20 ± 8.0^c^	16 ± 6.0^c^	18 ± 11^b^	18 ± 0.0^c^	22 ± 14^c^	—
*A. baumannii*	—	—	—	—	9.0 ± 0.0^d^	30 ± 16^b^
*B. pumilus*	12 ± 0.0^b^	5.0 ± 0.0^d^	3 ± 0.0^c^	11 ± 5.0^ab^	13 ± 8.0^ab^	—

*Note:* Values of the same column followed by different letters are significantly different at *p* ≤ 0.05.

Abbreviations: Ath, azithromycin; CD2, clindamycin; Fox, cefoxitin; IMI, imipenem; PG10, penicillin; T30, tetracycline.

*B. cereus* displayed susceptibility to cefoxitin and azithromycin, intermediate sensitivity to penicillin and tetracycline, but resistance to clindamycin, consistent with reports of ribosomal modification and efflux‐mediated resistance [96, 97]. This multidrug resistance pattern underscores the need for careful antibiotic selection, though targeted agents remain effective for controlling *B. cereus* in remediation contexts. *B. pumilus* exhibited resistance to clindamycin and penicillin, suggesting treatment challenges and the need for alternative or combination therapies. In contrast, *B. megaterium* was broadly susceptible to all tested antimicrobials, with the highest inhibition zone for penicillin (34 ± 20 mm), confirming its sensitivity and potential utility in bioremediation. *S. aureus* showed moderate or intermediate sensitivity, reflecting gene‐mediated variability in resistance [98–100].

In general, *B. megaterium* and *S. aureus* demonstrated significant susceptibility to specific antibiotics, positioning them as promising microbial agents for bioremediation. Their ability to degrade hydrocarbons while tolerating polluted environments enhances their environmental value. Understanding the resistance and susceptibility mechanisms of these isolates is critical for safe application, ensuring that remediation efforts do not inadvertently promote antibiotic resistance. Controlled use of these bacteria could strengthen ecosystem restoration in Mekelle while safeguarding public health.

## 4. Conclusion

This study demonstrates the strong potential of six bacterial isolates for oil spill remediation in Mekelle. *B. cereus* achieved the highest degradation rates across environments, while *S. aureus* and *A. baumannii* performed well in both soil and water. *B. pumilus* was highly effective in soil, *B. megaterium* maintained consistently high degradation across all conditions, and *P. aeruginosa* excelled in aquatic remediation. The isolates also exhibited diverse heavy metal tolerance, salt tolerance, and antibiotic susceptibility profiles. These findings highlight oil‐contaminated sites as reservoirs of efficient oil‐degrading bacteria, with significant implications for remediation in Mekelle and similar regions. Future work should focus on molecular characterization, enzyme identification, optimization of environmental parameters, and advanced metabolomic and metagenomic analyses to enhance their application.

## Author Contributions

Y.T.T.: conceptualization, data curation, formal analysis, investigation, methodology, project administration, and writing—original draft; D.B.S and G.G.: conceptualization, data curation, formal analysis, supervision, and writing—review and editing.

## Funding

This research was supported by Mekelle University under grant number MU/PG/006/2023.

## Ethics Statement

The authors have nothing to report.

## Consent

The authors have nothing to report.

## Conflicts of Interest

The authors declare no conflicts of interest.

## Data Availability

Data is available from the corresponding author upon reasonable request.
